# Comparative Gene-Expression Analysis of the Dental Follicle and Periodontal Ligament in Humans

**DOI:** 10.1371/journal.pone.0084201

**Published:** 2013-12-23

**Authors:** Hyo-Seol Lee, Jongeun Lee, Seong-Oh Kim, Je-Seon Song, Jae-Ho Lee, Syng-Ill Lee, Han-Sung Jung, Byung-Jai Choi

**Affiliations:** 1 Department of Pediatric Dentistry, College of Dentistry, Yonsei University, Seoul, Republic of Korea; 2 Oral Science Research Center, College of Dentistry, Yonsei University, Seoul, Republic of Korea; 3 Division in Oral Physiology, Department of Oral Biology, College of Dentistry, Yonsei University, Seoul, Republic of Korea; 4 Division in Anatomy & Developmental Biology, Department of Oral Biology, College of Dentistry, Yonsei University, Seoul, Republic of Korea; Instituto Butantan, Brazil

## Abstract

The human dental follicle partially differentiates into the periodontal ligament (PDL), but their biological functions are different. The gene-expression profiles of the dental follicle and PDL were compared using the cDNA microarray technique. Microarray analysis identified 490 genes with a twofold or greater difference in expression, 365 and 125 of which were more abundant in the dental follicle and PDL, respectively. The most strongly expressed genes in the dental follicle were those related to bone development and remodeling (*EGFL6*, *MMP8*, *FRZB*, and *NELL1*), apoptosis and chemotaxis (*Nox4*, *CXCL13*, and *CCL2*), and tooth and embryo development (*WNT2*, *PAX3*, *FGF7*, *AMBN*, *AMTN*, and *SLC4A4*), while in the PDL it was the tumor-suppressor gene *WIF1*. Genes related to bone development and remodeling (*STMN2*, *IBSP*, *BMP8A*, *BGLAP*, *ACP5*, *OPN*, *BMP3*, and *TM7SF4*) and wound healing (*IL1*, *IL8*, *MMP3*, and *MMP9*) were also more strongly expressed in the PDL than in the dental follicle. In selected genes, a comparison among cDNA microarray, real-time reverse-transcription polymerase chain reaction, and immunohistochemical staining confirmed similar relative gene expressions. The gene-expression profiles presented here identify candidate genes that may enable differentiation between the dental follicle and PDL.

## Introduction

The human dental follicle or dental sac is a loose ectomesenchymally derived connective tissue that surrounds the enamel organ and dental papilla of the developing tooth germ prior to eruption[1]. One of the biological functions of the dental follicle is the coordination of tooth eruption. Moreover, this tissue harbors progenitor cells for the periodontium[2], which, as the supporting tissue of the tooth, is composed of the periodontal ligament (PDL), alveolar bone, and the cementum that covers the tooth root surfaces[3]. The PDL tissue functions as a connector between the tooth and the alveolar jaw bone in the area surrounding the root surfaces[4]. Since the PDL originates from the dental follicle in tooth development, comparative gene-expression analyses may identify the unique features and specific functions of the two other dental tissues (i.e., alveolar bone and cementum).

The introduction of cDNA microarray technology, which has become one of the standard laboratory tools for gene-expression analysis, has made it possible to analyze thousands of genes simultaneously. This technique has been used in the following three ways in dental research:

1. To verify genes related to diseases or abnormal states, such as ameloblastoma[5], dental caries[6,7], and the PDL under mechanical stress *in* *vitro*[8].
2. To compare two similar normal tissues in order to determine subtle genetic differences, such as permanent PDL/deciduous PDL[8], fibroblast in the PDL/gingiva *in* *vitro*[9].
3. To compare two different tissues that share the same origin, such as odontoblast/pulp tissue[10].

Such research has revealed the gene expression of the human tooth. However, no comparative study has yet been made of the dental follicle and PDL.

The present study evaluated and compared the gene-expression profiles of the dental follicle and PDL in humans with the aim of explaining their functions, such as eruption coordination and stress resorption. This information may be applicable to clinical problems such as eruption disturbance, and to periodontal tissue engineering.

## Materials and Methods

### 1. Samples

The experimental protocol was approved by the Institutional Review Board of Yonsei University Dental Hospital, and written informed consent to participate in the study was obtained from all of the subjects and their parents (#2-2011-0055). PDL samples were obtained from healthy permanent premolars (*n*=11; 1 male and 4 females, aged 11–19 years) that had been extracted for orthodontic reasons. Dental follicles were obtained from children (*n*=4; 3 males and 1 female, aged 6–7 years) during the extraction of supernumerary teeth. The extracted teeth and dental follicles were immediately frozen and stored in liquid nitrogen. The PDL tissues were obtained carefully using sterile curettes from the middle-third. The PDL tissues and dental follicles were then immediately submerged in RLT buffer, the component of the RNeasy Fibrous Mini kit (Qiagen, CA, USA).

### 2. RNA isolation

The dental follicle and PDL tissues were homogenized separately using a Bullet Blender Bead (Next Advanced, NY, USA), and total RNA was extracted from the homogenates using the RNeasy Fibrous Mini kit (Qiagen) in accordance with the manufacturer’s instructions and as described previously[8]. The extracted RNA was eluted in 25 μl of sterile water and RNA concentrations were determined from absorbance values obtained at a wavelength of 260 nm as measured with the aid of a spectrophotometer (NanoDrop ND-1000, Thermo Scientific, IL, USA). The RNA samples used in this study had 260/280 nm ratios equal to or greater than 1.8.

### 3. cDNA microarray analysis

In the present study, global gene-expression analyses were performed using Affymetrix GeneChip Human Gene 1.0 ST oligonucleotide arrays (Affymetrix, CA, USA). The sample preparation was conducted in accordance with the manufacturer’s instructions and recommendations. The average amount of RNA isolated from dental follicles or PDL tissues was 1 μg. RNA quality was assessed with the aid of an Agilent 2100 bioanalyzer using the RNA 6000 Nano Chip system (Agilent Technologies, Amstelveen, The Netherlands), and the quantity was determined using the NanoDrop ND-1000 device (Thermo Scientific).

As recommended by the manufacturer’s protocol, 300 ng of total RNA from each sample was converted to double-strand cDNA. Using a random hexamer incorporating a T7 promoter, amplified RNA (cRNA) was generated from the double-stranded cDNA template though an *in vitro* transcription reaction and purified with the Affymetrix sample cleanup module. cDNA was regenerated through a random-primed reverse transcription (RT) using a dNTP mix containing dUTP. The cDNA was then fragmented by uracil-DNA glycosylase and apurinic/apyrimidinic endonuclease and restriction endonucleases, and end-labeled by terminal transferase reaction incorporating a biotinylated dideoxynucleotide. Fragmented end-labeled cDNA was hybridized to the GeneChip Human Gene 1.0 ST arrays for 16 hours at 45°C and 60 rpm, as described in the GeneChip Whole Transcript Sense Target Labeling Assay manual (Affymetrix). After hybridization, the chips were stained and washed in a GeneChip Fluidics Station 450 (Affymetrix) and scanned using a GeneChip Array scanner 3000 G7 (Affymetrix); the image data were extracted using Affymetrix Command Console software version 1.1 (Affymetrix). The raw file generated through this procedure contained expression intensity data and was used for the next step of the analysis.

### 4. Microarray data analysis

The expression data were generated by Affymetrix Expression Console software version 1.1 (Affymetrix). The Robust Multi-array Average (RMA) algorithm implemented in the Affymetrix Expression Console software was used to normalize the data. A one-way ANOVA was performed on the RMA expression values to determine whether genes were differentially expressed between the three groups. A multiple-testing correction was applied to the *p* values of the *F*-statistics to adjust the false discovery rate (Benjamini and Hochberg, 1995). Genes with adjusted *F*-statistic *p* values of <0.05 were extracted. Microarray analysis identified 490 genes with expression differences of twofold or greater, 365 and 125 of which were more abundant in the dental follicle and PDL tissue, respectively. However, only strongly expressed genes in the dental follicles or PDL that differed by more than 4- or 2.5-fold from the signal of the control and each test group, respectively, were selected for further study. In order to classify the coexpression gene group with a similar expression pattern, hierarchical clustering and K-mean clustering were performed using MultiExperiment Viewer software version 4.4 (www.tm4.org; Dana-Farber Cancer Institute, MA, USA). The Web-based tool Database for Annotation, Visualization, and Integrated Discovery (DAVID) was used for the biological interpretation of differentially expressed genes. These genes were classified based on data on gene function in the gene ontology of the Kyoto Encyclopedia of Genes and Genomes (KEGG) pathway database (http://david.abcc.ncifcrf.gov/home.jsp).

This microarray data set was approved by the Gene Expression Omnibus (http://www.ncbi.nlm.gov/geo/); its GEO accession number is GSE51342.

### 5. Quantitative reverse-transcription polymerase chain reaction

The single-stranded cDNA required for the polymerase chain reaction (PCR) analysis was produced using 500 ng of extracted total RNA as a template for RT (Superscript III Reverse Transcriptase and random primer, Invitrogen, UK). The RT reaction was performed at 65°C for 5 minutes, followed by 25°C for 5 minutes, 50°C for 1 hour, and 70°C for 15 minutes to inactivate the activity of the reverse transcriptase. The synthesized cDNA was diluted at 10:1 in distilled water and used as a template for quantitative RT-PCR, which was performed using the ABI7300 RT-PCR system (Applied Biosystems, Warrington, UK). Samples of 25 μl containing 1× Universal TaqMan Master Mix (4369016, Applied Biosystems), PCR primers at a concentration of 0.9 μM, and the diluted cDNA were prepared in triplicate. The amplification conditions were 50°C for 2 minutes and 95°C for 10 minutes followed by 40 cycles of 95°C for 15 seconds and 60°C for 1 minute. The following TaqMan gene-expression assay primers (Applied Biosystems) were used: *AMTN*, *CD36*, *CXCL13*, *DMP1*, *EGFL6*, *MMP8*, *MMP9*, *WIF1*, and 18S rRNA. ABI 7300 SDS 1.3.1 software (Applied Biosystems) was used to record the fluorescence intensity of the reporter and quencher dyes; the results are plotted versus time, quantified as the cycle number. A precise quantification of the initial target was obtained by examining the amplification plots during the early log phase of product accumulation above background [the threshold cycle (Ct) number]. Ct values were subsequently used to determine ΔCt values (ΔCt=Ct of the gene minus Ct of the 18S rRNA control), and differences in Ct values were used to quantify the relative amount of PCR product, expressed as the relative change by applying the equation 2^–ΔCt^.

### 6. Immunohistochemical staining

For immunohistochemical (IHC) staining, permanent teeth were fixed in 10% buffered formalin (Sigma, MO, USA) for 1 day and then decalcified with 10% EDTA (pH 7.4; Fisher Scientific, TX, USA) for 8 weeks. The dental follicles and the permanent teeth were embedded in paraffin, and sectioned at a thickness of 3 μm. Specimens were subjected to IHC staining with antihuman AMTN (rabbit polyclonal, diluted 1:100; Ab122312, Abcam, Cambridge, UK), CXCL13 (BCA1, rabbit polyclonal, diluted 1:200; Ab112521, Abcam), DMP1 (rabbit polyclonal, diluted 1:100; Ab82351, Abcam), WIF1 (rabbit polyclonal, diluted 1:100; Ab71204, Abcam), and MMP9 (rabbit polyclonal, diluted 1:800; Ab38898, Abcam). The endogenous peroxidase activity was quenched by the addition of 3% hydrogen peroxide. Sections were incubated in 5% bovine serum albumin to block nonspecific binding. The primary antibodies in which the sections were incubated overnight were diluted so as to optimize the staining. After incubation, ready to use EnVision+ System HRP-labeled polymer antirabbit (K4003, ready to use; Dako North America, CA, USA) was applied for 20 minutes. Color development was performed using labeled streptavidin biotin kits (Dako) following the manufacturer’s instructions. The sections were counterstained with Gill’s hematoxylin (Sigma). Control sections were treated in the same manner except for the treatment with primary antibodies.

## Results

### 1. Dental follicle and PDL gene-expression profiles

Complementary DNA microarray technology was used to compare multiple gene-expression profiles representative of the dental follicle and PDL tissues. The results indicated that the expression levels of 490 out of 33,297 (1.49%) genes had changed by at least twofold in one tissue type relative to the other. In the dental follicle, the expression levels of 365 genes were at least double those in the PDL, while in the latter, the expression levels of 125 genes were at least double those in dental follicle tissue. The total data distribution and frequency were confirmed in density and box plots and in scatter plots of the standardized log intensity ratio versus the average intensity (Figure S1). Ultimately, 108 genes were analyzed further. In dental follicle tissue, 55 genes were up-regulated by at least fourfold relative to PDL tissue (Table 1), while 53 genes were up-regulated by at least 2.5-fold in the PDL relative to dental follicle tissue (Table 2).

**Table 1:** Up-regulated genes in the dental follicle tissue (compared to periodontal ligament tissue).

**Name**	**Gene symbol**	**Fold change**	**Gene Accession**	**Cytoband**
ameloblastin (enamel matrix protein)	AMBN	94.88	NM_016519	4q21
amelotin	AMTN	22.23	NM_212557	4q13.3
EGF-like-domain, multiple 6	EGFL6	17.76	NM_015507	Xp22
microfibrillar associated protein 5	MFAP5	14.29	NM_003480	12p13.1-p12.3
leucine-rich repeat-containing G protein-coupled receptor 5	LGR5	12.93	NM_003667	12q22-q23
neuronal growth regulator 1	NEGR1	12.85	NM_173808	1p31.1
coagulation factor II (thrombin) receptor-like 2	F2RL2	10.99	NM_004101	5q13
hydroxyprostaglandin dehydrogenase 15-(NAD)	HPGD	10.66	NM_000860	4q34-q35
gamma-aminobutyric acid (GABA) A receptor, alpha 3	GABRA3	9.60	NM_000808	Xq28
PDZ domain containing ring finger 4	PDZRN4	8.61	NM_013377	12q12
frizzled-related protein	FRZB	8.26	NM_001463	2qter
statherin	STATH	7.57	NM_003154	4q13.3
triadin	TRDN	7.31	NM_006073	6q22.31
chemokine (C-X-C motif) ligand 13	CXCL13	7.21	NM_006419	4q21
leucine rich repeat neuronal 1	LRRN1	7.10	NM_020873	3p26.2
C1q and tumor necrosis factor related protein 3	C1QTNF3	7.09	NM_181435	5p13
spondin 1, extracellular matrix protein	SPON1	6.66	NM_006108	11p15.2
ectonucleotidepyrophosphatase/phosphodiesterase 5 (putative)	ENPP5	6.52	NM_021572	6p21.1-p11.2
epithelial cell adhesion molecule	EPCAM	6.41	NM_002354	2p21
solute carrier family 4, sodium bicarbonate cotransporter, member 4	SLC4A4	6.33	NM_001098484	4q21
ALX homeobox 1	ALX1	6.29	NM_006982	12q21.31
NADPH oxidase 4	NOX4	6.23	NM_016931	11q14.2-q21
fibroblast growth factor 7	FGF7	5.75	NM_002009	15q21.2
matrix metallopeptidase 8 (neutrophil collagenase)	MMP8	5.58	NM_002424	11q22.3
small nucleolar RNA, C/D box 114-26	SNORD114-26	5.58	NR_003219	14q32
sarcoglycan, zeta	SGCZ	5.49	NM_139167	8p22
	---	5.38	---	---
hyaluronan and proteoglycan link protein 1	HAPLN1	5.12	NM_001884	5q14.3
CD24 molecule	CD24	4.94	NM_013230	6q21
transient receptor potential cation channel, subfamily A, member 1	TRPA1	4.86	NM_007332	8q13
carboxypeptidase X (M14 family), member 2	CPXM2	4.85	NM_198148	10q26.13
paternally expressed 10	PEG10	4.75	NM_015068	7q21
sema domain, immunoglobulin domain (Ig), short basic domain, secreted, (semaphorin) 3A	SEMA3A	4.67	NM_006080	7p12.1
flavin containing monooxygenase 3	FMO3	4.66	NM_006894	1q24.3
wingless-type MMTV integration site family member 2	WNT2	4.65	NM_003391	7q31.2
thrombospondin 4	THBS4	4.62	NM_003248	5q13
pappalysin 2	PAPPA2	4.59	NM_020318	1q23-q25
G protein-coupled receptor 64	GPR64	4.53	NM_001079858	Xp22.13
elongation of very long chain fatty acids (FEN1/Elo2, SUR4/Elo3, yeast)-like 2	ELOVL2	4.51	NM_017770	6p24.2
calcium channel, voltage-dependent, alpha 2/delta subunit 3	CACNA2D3	4.48	NM_018398	3p21.1
complement component 3	C3	4.46	NM_000064	19p13.3-p13.2
collagen, type XIV, alpha 1	COL14A1	4.36	NM_021110	8q23
Ras protein-specific guanine nucleotide-releasing factor 2	RASGRF2	4.31	NM_006909	5q13
activated leukocyte cell adhesion molecule	ALCAM	4.30	NM_001627	3q13.1
neural cell adhesion molecule 2	NCAM2	4.23	NM_004540	21q21.1
dickkopf homolog 2 (Xenopuslaevis)	DKK2	4.22	NM_014421	4q25
hemicentin 1	HMCN1	4.20	NM_031935	1q25.3-q31.1
core 1 synthase, glycoprotein-N-acetylgalactosamine 3-beta-galactosyltransferase, 1	C1GALT1	4.17	NM_020156	7p14-p13
NEL-like 1 (chicken)	NELL1	4.16	NM_006157	11p15.1
chromosome 12 open reading frame 75	C12orf75	4.14	NM_001145199	12q23.3
odz, odd Oz/ten-m homolog 2 (Drosophila)	ODZ2	4.13	AK302302	5q34
paired box 3	PAX3	4.09	NM_181458	2q35-q37|2q35
solute carrier family 38, member 1	SLC38A1	4.08	NM_030674	12q13.11
gremlin 1	GREM1	4.04	NM_013372	15q13.3
solute carrier family 1 (glial high affinity glutamate transporter), member 3	SLC1A3	4.01	NM_004172	5p13
Kallmann syndrome 1 sequence	KAL1	4.00	NM_000216	Xp22.32
microfibrillar-associated protein 4	MFAP4	3.97	NM_001198695	17p11.2
dermatan sulfate epimerase-like	DSEL	3.96	NM_032160	18q22.1
laminin, alpha 2	LAMA2	3.95	NM_000426	6q22-q23
vesicle amine transport protein 1 homolog (T. californica)-like	VAT1L	3.91	NM_020927	16q23.1
chemokine (C-C motif) ligand 2	CCL2	3.88	NM_002982	17q11.2-q12

**Table 2:** Up-regulated genes in the periodontal ligament tissue (compared to dental follicle).

**Name**	**Gene symbol**	**Fold change**	**Gene access**	**cytoband**
WNT inhibitory factor 1	WIF1	12.63	NM_007191	12q14.3
dentin matrix acidic phosphoprotein 1	DMP1	10.67	NM_004407	4q21
acid phosphatase 5, tartrate resistant	ACP5	6.78	NM_001111035	19p13.3-p13.2
small proline-rich protein 2A	SPRR2A	6.60	NM_005988	1q21-q22
bone morphogenetic protein 8a	BMP8A	6.15	NM_181809	1p34.3
transmembrane 4 L six family member 19	TM4SF19	6.12	NM_138461	3q29
tenascin N	TNN	6.02	NM_022093	1q23-q24
stathmin-like 2	STMN2	5.57	NM_007029	8q21.13
bone gamma-carboxyglutamate (gla) protein	BGLAP	5.10	NM_199173	1q25-q31
signal peptide, CUB domain, EGF-like 3	SCUBE3	4.55	NM_152753	6p21.3
CD36 molecule (thrombospondin receptor)	CD36	4.54	NM_001001548	7q11.2
ATPase, H+ transporting, lysosomal 38kDa, V0 subunit d2	ATP6V0D2	4.25	NM_152565	---
small Cajal body-specific RNA 17	SCARNA17	4.21	NR_003003	18q21.1
matrix metallopeptidase 9 (gelatinase B, 92kDa gelatinase, 92kDa type IV collagenase)	MMP9	4.20	NM_004994	20q11.2-q13.1
multiple EGF-like-domains 10	MEGF10	4.14	NM_032446	5q33
interleukin 1, beta	IL1B	4.11	NM_000576	2q14
G protein-coupled receptor 109B	GPR109B	4.10	NM_006018	12q24.31
interleukin 8	IL8	4.07	NM_000584	4q13-q21
---	---	3.98	---	---
anoctamin 5	ANO5	3.94	NM_213599	11p14.3
integrin-binding sialoprotein	IBSP	3.91	NM_004967	4q21.1
matrix metallopeptidase 3 (stromelysin 1, progelatinase)	MMP3	3.80	NM_002422	11q22.3
matrix extracellular phosphoglycoprotein	MEPE	3.73	NM_001184694	4q21.1
interferon, gamma-inducible protein 30	IFI30	3.61	NM_006332	19p13.1
creatine kinase, brain	CKB	3.46	NM_001823	14q32
integrin, alpha 10	ITGA10	3.42	NM_003637	1q21
small Cajal body-specific RNA 17	SCARNA17	3.38	NR_003003	18q21.1
---	---	3.29	---	---
BMP and activin membrane-bound inhibitor homolog (Xenopuslaevis)	BAMBI	3.19	NM_012342	10p12.3-p11.2
pannexin 3	PANX3	3.18	NM_052959	11q24.2
solute carrier family 37 (glycerol-3-phosphate transporter), member 2	SLC37A2	3.18	NM_198277	11q24.2
transcription factor AP-2 beta (activating enhancer binding protein 2 beta)	TFAP2B	3.14	NM_003221	6p12
bone morphogenetic protein 3	BMP3	3.09	NM_001201	4q21
distal-less homeobox 1	DLX1	2.98	NM_178120	2q32
interferon induced transmembrane protein 5	IFITM5	2.97	NM_001025295	11p15.5
bone morphogenetic protein 8b	BMP8B	2.94	NM_001720	1p35-p32
myelin protein zero	MPZ	2.93	NM_000530	1q23.3
MAM domain containing 2	MAMDC2	2.91	NM_153267	9q21.12
dachsous 2 (Drosophila)	DCHS2	2.90	NM_017639	4q31.3
chondroadherin	CHAD	2.85	NM_001267	17q21.33
serpin peptidase inhibitor, clade E (nexin, plasminogen activator inhibitor type 1), member 1	SERPINE1	2.83	NM_000602	7q21.3-q22
selectin E	SELE	2.77	NM_000450	1q22-q25
transmembrane 7 superfamily member 4	TM7SF4	2.77	NM_030788	8q23
aquaporin 9	AQP9	2.76	NM_020980	15q
protein tyrosine phosphatase, receptor type, f polypeptide (PTPRF), interacting protein (liprin), alpha 2	PPFIA2	2.76	NM_003625	12q21.31
phospholipase A2, group IIA (platelets, synovial fluid)	PLA2G2A	2.75	NM_000300	1p35
lysophosphatidic acid receptor 3	LPAR3	2.71	NM_012152	1p22.3
rhomboid, veinlet-like 2 (Drosophila)	RHBDL2	2.69	NM_017821	1p34.3
---	---	2.67	---	---
discs, large (Drosophila) homolog-associated protein 5	DLGAP5	2.64	NM_014750	14q22.3
major histocompatibility complex, class II, DQ alpha 1	HLA-DQA1	2.60	NM_002122	6p21.3
von Willebrand factor D and EGF domains	VWDE	2.58	NM_001135924	7p21.3
family with sequence similarity 40, member B	FAM40B	2.57	NM_020704	7q32.1
chemokine (C-X-C motif) receptor 1	CXCR1	2.57	NM_000634	2q35
retinol binding protein 4, plasma	RBP4	2.52	NM_006744	10q23-q24
G protein-coupled receptor 109A	GPR109A	2.50	NM_177551	12q24.31
microRNA 487a	MIR487A	2.50	NR_030162	14q32.31

### 2. Gene-ontology analysis

**Figure 1:**
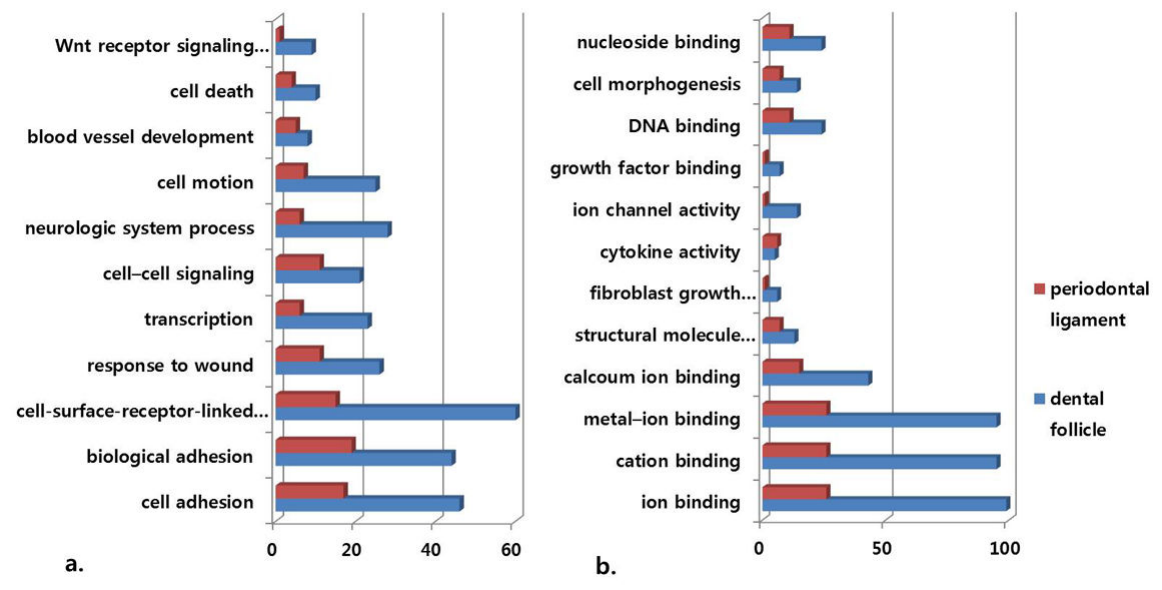
Gene-ontology analysis of the periodontal ligament(PDL) and the dental follicle. a. Main categories of genes expressed specifically in the dental follicle and periodontal ligament (PDL) tissues on the basis of their biological processes. b. Main categories of genes expressed specifically in dental follicle and PDL tissues on the basis of their molecular function (*x*-axis: number of involved genes, *F*-statistic *p*<0.05).

In order to identify the biological functions and features of the selected genes, the expressed data sets were organized into Gene Ontology Consortium (GO) grouping using the DAVID Web-based tool. These genes were then classified based on information regarding gene function using gene ontology from the KEGG pathway database. Figure 1 show GO classes with *F*-statistic *p* values of <0.05 for the two data sets analyzed.

Biological process analysis revealed that genes related to cell surface receptor-linked signal transduction, cell adhesion, biological adhesion, neurologic system processes, and Wnt receptor signaling pathway were more strongly expressed in dental follicle tissue than in the PDL. Moreover, molecular function analysis revealed that genes related to ion binding, cation binding, metal ion binding, and calcium ion binding were more strongly expressed in dental follicle tissue than in the PDL.

### 3. Quantitative RT-PCR

Quantitative RT-PCR analysis was performed to verify the results obtained through cDNA microarray. The eight genes with expression levels differing by at least twofold between dental follicle tissue and the PDL were selected (Table S1). For the statistical analysis, the unpaired *t*-test (α<0.05) was performed to correlate the relative change with differential expression as detected by PCR. *AMTN*, *EGFL-6*, *CXCL13*, and *MMP8* were up-regulated in dental follicle tissue relative to the PDL, while *DMP1*, *WIF1* and *CD36* were up-regulated in PDL tissue relative to the dental follicle (Table 3). The expression of *MMP9* did not differ between dental follicle tissue and PDL tissue. These results are consistent with the microarray results.

**Table 3:** The relative difference in gene mRNA expression in dental follicle and periodontal ligament tissue.

Gene	Relative Gene Expression (mean±SD)	
	Dental Follicle Tissues	Periodontal Ligament Tissues
AMTN**	2741.88±245.81	1
EGFL-6**	265.40±79.19	1
CXCL13**	12.15±1.23	1
MMP8**	3.48±0.70	1
DMP1**	1	51.59±14.64
WIF1**	1	233.17±24.40
CD36**	1	5.32±1.26
*MMP9*	1	1.34±0.26

^**^ *p*<0.01

### 4. IHC staining

**Figure 2:**
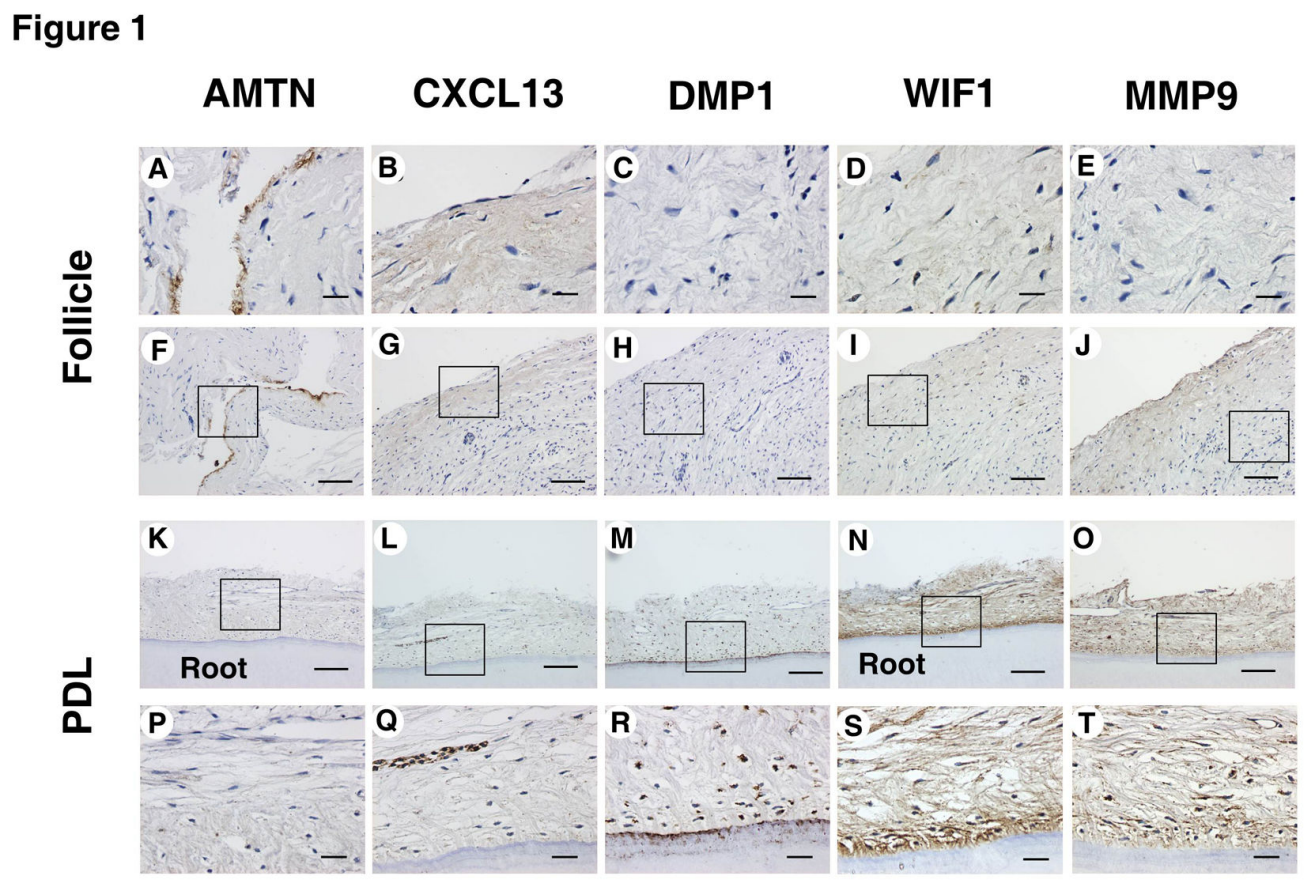
Immunohistochemical (IHC) staining of dental follicle and permanent PDL tissues. (**A**, **F**) IHC staining for AMTN in the dental follicle and (**K**, **P**) permanent PDL tissues. (**B**, **G**) IHC staining for CXCL13 in the dental follicle and (**L**, **Q**) permanent PDL tissue. (**C**, **H**) IHC staining for DMP1 in the dental follicle and (**M**, **R**) permanent PDL tissue. (**D**, **I**) IHC staining for WIF1 in the dental follicle and (**N**, **S**) permanent PDL tissue. (**E**, **J**) IHC staining for MMP9 in the dental follicle and (**O**, **T**) permanent PDL tissue. (Scale bars: 20 μm in **A–E** and **P–T**; 100 μm in F–O.)

The following five genes were the targets of the IHC study: *AMTN*, *CXCL13*, *DMP1*, *WIF1*, and *MMP9* (Figure 2). *AMTN* staining was found only in the reduced enamel epithelium of the dental follicle. *CXCL13* was broadly stained in the outer area of the dental follicles, and in Hertwig’s epithelial root sheath (HERS). *DMP1* was not stained in dental follicle tissue, but was found around the cementoblast layer. Similarly, *WIF1* was not stained in dental follicle tissue but was strongly stained in permanent PDL tissues, especially in the cementoblast layer. *MMP9* was broadly stained in the outer area of the dental follicle but also in all layers of permanent PDL tissue. These results are consistent with those of the cDNA microarray analysis at the protein level.

## Discussion

The cDNA microarray results indicate that the expression levels of 490 out of 33,297 (1.49%) genes had changed by at least twofold in one tissue type relative to the other. Although this finding is not dissimilar to that of McLachlan et al.[7] in dental caries (445/15,000, 2.96%), Song [8] reported that only 51 of >20,000 genes (≤0.25%) were differentially expressed between the PDLs of permanent teeth and deciduous teeth. Differences in the homogeneity between dental follicle and PDL tissue from different tooth types may explain this discrepancy, with PDL tissues from different tooth types possibly being more homogeneous than the dental follicle.

In general, genes associated with bone development and remodeling (*EGFL6*, *MMP8*, *FRZB*, and *NELL1*), apoptosis and chemotaxis (*Nox4*, *CXCL13*, and *CCL2*), and tooth and embryo development (*WNT2*, *PAX3*, *FGF7*, *AMBN*, *AMTN*, and *SLC4A4*) were more strongly expressed in the dental follicle than in the PDL. These genes are thought to be associated with the biological function of dental follicle tissue, such as eruption coordination.

In detail, bone remodeling requires an intimate cross-talk between osteoclasts and osteoblasts. *EGFL6*, a member of the epidermal growth factor repeat superfamily of proteins, induces angiogenesis via a paracrine mechanism in which *EGFL6* is expressed in osteoblast-like cells but promotes the migration and angiogenesis of endothelial cells [11]. *MMP8* is involved in the breakdown of extracellular matrix in normal physiological processes, such as tissue remodeling[12]. *FRZB* (frizzled motif associated with bone development) plays an important role in bone development [13]. *NELL1* promotes osteoblast cell differentiation and mineralization. *Runx2*, *Osterix*, and *NELL1* promote crosstalk during osteogenesis [14].

*Nox4* promotes the apoptosis of oral epithelium at the eruption site and so is thought to be related to the tooth-eruption mechanism [15]. *CXCL13* [chemokine (C-X-C motif) ligand 13] promotes the migration of B lymphocytes and is strongly expressed in the liver, spleen, and lymph nodes [16]. The number of *CXCL13* cells is reportedly significantly higher in periodontitis, suggesting that this condition is associated with alveolar bone resorption. *CCL2* (*MCP-1*) is a well-known chemokine for monocytes and a prime candidate for recruiting the osteoclast precursors into dental follicle tissue [17].

Genes associated with tooth and embryo development were also significantly more strongly expressed in the dental follicle than in the PDL. *WNT2* is a member of the WNT gene family, and the Wnt pathway is crucial for tooth development, embryogenesis, and odontoblast and ameloblast differentiation [18]. Previous studies found that Wnt signaling inhibits cell differentiation and promotes cell proliferation in cementoblasts *in vitro* [19]. Furthermore, the Wnt signaling pathways may participate in the formation of the tooth eruption passage [15].

The tumor-suppressor gene *WIF1* was the most strongly expressed in the PDL. In addition, genes associated with wound healing (*IL1*, *IL8*, *MMP3*, and *MMP9*) and bone development and remodeling (*STMN2*, *IBSP*, *BMP8A*, *BGLAP*, *ACP5*, *OPN*, *BMP3*, *TM7SF4*) were up-regulated relative to the dental follicle.

*WIF1* was the most strongly expressed gene in the PDL, which means that, conversely, the Wnt signal would have been strongly expressed in dental follicle tissue. WNT2 and DKK2 are associated with WNT signaling, and the genes encoding both were strongly expressed in the dental follicle in this study. The Wnt signaling pathway has been implicated in a wide range of biological process, from maintaining the pluripotentiality of stem cells to the induction of specific tissues and organs during development. *WIF1* functions as a tumor-suppressor gene, and it has been shown to be epigenetically silenced in various cancers[20]. Genes related to wound healing were significantly strongly expressed. *IL1* and *IL8* are important mediators of the inflammatory response and play an important role in the induction of osteoclastic bone resorption during orthodontic tooth movement [21]. *MMP3* and *MMP9* are regulated by fibroblasts in the PDL and are induced in fibroblasts and immune cells that are recruited in the pathologic situation [22].

Genes associated with bone development and remodeling were significantly more strongly expressed in the PDL than in the dental follicle. *STMN2* is a novel marker of osteogenesis and osteoblasts [23]. IBSP (integrin-binding sialoprotein) is a major structural protein of the bone matrix [24]. *BMP8A* is related to osteogenesis and skeletal development [24,25]. *BGLAP* (osteocalcin) was first identified in the mineralized matrix of bone and is associated with bone development [26]. *ACP5* (TRAP) exhibits optimal activity in acidic conditions and is strongly expressed by osteoclasts, activated macrophages, and neurons. *OPN* and bone sialoprotein are strongly associated with TRAP when phosphorylated [27]. *BMP3* is a major structural protein of the bone matrix and osteoclast activity in fractured sites. This gene is a member of the transforming growth factor beta superfamily and is localized to the latest stage of periodontium formation by inhibiting the signaling provided by other bone morphogenetic proteins [28]. *TM7SF4* is related to osteoclastogenesis and the regulation of osteoclast fusion [29].

Quantitative RT-PCR analyses were carried out to verify our cDNA microarray results. *AMTN*, *EGFL-6*, *CXCL13*, and *MMP8* were up-regulated in dental follicle tissue relative to the PDL, while *DMP1*, *WIF1* and *CD36* were up-regulated in the PDL tissue relative to the dental follicle. These findings are consistent with the microarray results.

To better understand the roles of the differentially expressed genes identified by the microarray analyses between dental follicle and PDL tissues in humans, their cellular origins were identified using IHC analysis. *AMTN* was found only in the reduced enamel epithelium of dental follicle tissue. *CXCL13* was broadly stained in the outer area of the dental follicle and in HERS. *DMP1* was not stained in the dental follicle, but was found around the cementoblast layer. *WIF1* was not stained in dental follicles, but was strongly stained in permanent PDL tissue, especially the cementoblast layer. *MMP9* was broadly stained in the outer area of the dental follicles, but also strongly in all layers of permanent PDL tissue (Figure 2). These results are consistent with those of the cDNA microarray analysis at the protein level.

In conclusion, genes associated with bone development and remodeling were strongly expressed in both PDL and dental follicle tissues, and *in vivo* would induce tooth eruption or tooth movement. The two tissues also expressed specific genes related to their functions. Interestingly, *WIF1*, which was strongly expressed in the PDL, is thought to represent a counter example of the pluripotentiality of the stem cells of the dental follicle. The findings of this study are expected to contribute to future research in the field of clinical problems such as eruption disturbance, and to research in periodontal tissue engineering by demonstrating the differences between the PDL and dental follicle tissues at the molecular biological level.

## Supporting Information

Figure S1 **Scatter plots of the differences in the log intensities versus the average log intensity between three dental follicle tissue samples and three PDL tissue samples.** In each plot the *x*-axis (A) is 0.5 × [log_2_(case)+log_2_ (control)] and the *y*-axis (M) is log_2_ (case/control). The data of all plots were normally distributed.
(TIF)

Table S1 **Quantitative RT-PCR primers used in this study.**
(DOCX)

## References

[1] ten Cate AR (1997) The development of the periodontium-a largely ectomesenchymally derived unit. Periodontol 2000 13: 9-19. doi:10.1111/j.1600-0757.1997.tb00093.x. PubMed: 9567921. 9567921

[2] Morsczeck C, Schmalz G, Reichert TE, Völlner F, Galler K et al. (2008) Somatic stem cells for regenerative dentistry. Clin Oral Investig 12: 113-118. doi:10.1007/s00784-007-0170-8. PubMed: 18172700. 18172700

[3] Saugspier M, Felthaus O, Viale-Bouroncle S, Driemel O, Reichert TE et al. (2010) The differentiation and gene expression profile of human dental follicle cells. Stem Cells Dev 19: 707-717. doi:10.1089/scd.2010.0027. PubMed: 20491563. 20491563

[4] McCulloch CA (2006) Proteomics for the periodontium: current strategies and future promise. Periodontol 2000 40: 173–83. PubMed: 16398693. 16398693 10.1111/j.1600-0757.2005.00135.x

[5] Heikinheimo K, Jee KJ, Niini T, Aalto Y, Happonen RP et al. (2002) Gene expression profiling of ameloblastoma and human tooth germ by means of a cDNA microarray. J Dent Res 81: 525-530. doi:10.1177/154405910208100805. PubMed: 12147741. 12147741

[6] Pääkkönen V, Ohlmeier S, Bergmann U, Larmas M, Salo T et al. (2005) Analysis of gene and protein expression in healthy and carious tooth pulp with cDNA microarray and two-dimensional gel electrophoresis. Eur J Oral Sci 113: 369-379. doi:10.1111/j.1600-0722.2005.00237.x. PubMed: 16202023. 16202023

[7] McLachlan JL, Smith AJ, Bujalska IJ, Cooper PR (2005) Gene expression profiling of pulpal tissue reveals the molecular complexity of dental caries. Biochim Biophys Acta 1741: 271-281. doi:10.1016/j.bbadis.2005.03.007. PubMed: 15869869. 15869869

[8] Song JS, Hwang DH, Kim SO, Jeon M, Choi BJ et al. (2013) Comparative gene expression analysis of the human periodontal ligament in deciduous and permanent teeth. PLOS ONE 8: e61231. doi:10.1371/journal.pone.0061231. PubMed: 23593441. 23593441 PMC3620385

[9] Han X, Amar S (2002) Identification of genes differentially expressed in cultured human periodontal ligament fibroblasts vs. human gingival fibroblasts by DNA microarray analysis. J Dent Res 81: 399-405. doi:10.1177/154405910208100609. PubMed: 12097432. 12097432

[10] Pääkkönen V, Vuoristo JT, Salo T, Tjäderhane L (2008) Comparative gene expression profile analysis between native human odontoblasts and pulp tissue. Int Endod J 41: 117-127. PubMed: 18005044. 18005044 10.1111/j.1365-2591.2007.01327.x

[11] Chim SM, Qin A, Tickner J, Pavlos N, Davey T et al. (2011) EGFL6 promotes endothelial cell migration and angiogenesis through the activation of extracellular signal-regulated kinase. J Biol Chem 286: 22035-22046. doi:10.1074/jbc.M110.187633. PubMed: 21531721. 21531721 PMC3121348

[12] Lee YH, Nahm DS, Jung YK, Choi JY, Kim SG et al. (2007) Differential gene expression of periodontal ligament cells after loading of static compressive force. J Periodontol 78: 446-452. doi:10.1902/jop.2007.060240. PubMed: 17335367. 17335367

[13] Qu Y, Li JF, Cai Q, Wang YW, Gu QL et al. (2008) Over-expression of FRZB in gastric cancer cell suppresses proliferation and induces differentiation. J Cancer Res Clin Oncol 134: 353-364. doi:10.1007/s00432-007-0291-0. PubMed: 17680269. 17680269 PMC12161657

[14] Chen F, Zhang X, Sun S, Zara JN, Zou X et al. (2011) NELL-1, an osteoinductive factor, is a direct transcriptional target of Osterix. PLOS ONE 6: e24638. doi:10.1371/journal.pone.0024638. PubMed: 21931789. 21931789 PMC3172249

[15] Moriguchi M, Yamada M, Miake Y, Yanagisawa T (2010) Transforming growth factor beta inducible apoptotic cascade in epithelial cells during rat molar tooth eruptions. Anat Sci Int 85: 92-101. doi:10.1007/s12565-009-0061-y. PubMed: 19779767. 19779767

[16] Amft N, Curnow SJ, Scheel-Toellner D, Devadas A, Oates J et al. (2001) Ectopic expression of the B cell-attracting chemokine BCA-1 (CXCL13) on endothelial cells and within lymphoid follicles contributes to the establishment of germinal center-like structures in Sjogren's syndrome. Arthritis Rheum 44: 2633-2641. doi:10.1002/1529-0131(200111)44:11. PubMed: 11710719. 11710719

[17] Rollins BJ, Morrison ED, Stiles CD (1988) Cloning and expression of JE, a gene inducible by platelet-derived growth factor and whose product has cytokine-like properties. Proc Natl Acad Sci U S A 85: 3738-3742. doi:10.1073/pnas.85.11.3738. PubMed: 3287374. 3287374 PMC280293

[18] Suomalainen M, Thesleff I (2010) Patterns of Wnt pathway activity in the mouse incisor indicate absence of Wnt/beta-catenin signaling in the epithelial stem cells. Dev Dyn 239: 364-372. PubMed: 19806668. 19806668 10.1002/dvdy.22106

[19] Silvério KG, Davidson KC, James RG, Adams AM, Foster BL et al. (2012) Wnt/beta-catenin pathway regulates bone morphogenetic protein (BMP2)-mediated differentiation of dental follicle cells. J Periodontal Res 47: 309-319. doi:10.1111/j.1600-0765.2011.01433.x. PubMed: 22150562. 22150562 PMC3865600

[20] Chan SL, Cui Y, van Hasselt A, Li H, Srivastava G et al. (2007) The tumor suppressor Wnt inhibitory factor 1 is frequently methylated in nasopharyngeal and esophageal carcinomas. Lab Invest 87: 644-650. doi:10.1038/labinvest.3700547. PubMed: 17384664. 17384664

[21] Baba S, Kuroda N, Arai C, Nakamura Y, Sato T (2011) Immunocompetent cells and cytokine expression in the rat periodontal ligament at the initial stage of orthodontic tooth movement. Arch Oral Biol 56: 466-473. doi:10.1016/j.archoralbio.2010.11.010. PubMed: 21193170. 21193170

[22] Kubota T, Itagaki M, Hoshino C, Nagata M, Morozumi T et al. (2008) Altered gene expression levels of matrix metalloproteinases and their inhibitors in periodontitis-affected gingival tissue. J Periodontol 79: 166-173. doi:10.1902/jop.2008.070159. PubMed: 18166107. 18166107

[23] Chiellini C, Grenningloh G, Cochet O, Scheideler M, Trajanoski Z et al. (2008) Stathmin-like 2, a developmentally-associated neuronal marker, is expressed and modulated during osteogenesis of human mesenchymal stem cells. Biochem Biophys Res Commun 374: 64-68. doi:10.1016/j.bbrc.2008.06.121. PubMed: 18611392. 18611392

[24] Chen J, McCulloch CA, Sodek J (1993) Bone sialoprotein in developing porcine dental tissues: cellular expression and comparison of tissue localization with osteopontin and osteonectin. Arch Oral Biol 38: 241-249. doi:10.1016/0003-9969(93)90034-J. PubMed: 8489418. 8489418

[25] Paic F, Igwe JC, Nori R, Kronenberg MS, Franceschetti T et al. (2009) Identification of differentially expressed genes between osteoblasts and osteocytes. Bone 45: 682-692. doi:10.1016/j.bone.2009.06.010. PubMed: 19539797. 19539797 PMC2731004

[26] Kayed H, Bekasi S, Keleg S, Michalski CW, Giese T et al. (2007) BGLAP is expressed in pancreatic cancer cells and increases their growth and invasion. Mol Cancer 6: 83. doi:10.1186/1476-4598-6-83. PubMed: 18163903. 18163903 PMC2245975

[27] Ek-Rylander B, Flores M, Wendel M, Heinegård D, Andersson G (1994) Dephosphorylation of osteopontin and bone sialoprotein by osteoclastic tartrate-resistant acid phosphatase. Modulation of osteoclast adhesion in vitro. J Biol Chem 269: 14853-14856. PubMed: 8195113. 8195113

[28] Kémoun P, Laurencin-Dalicieux S, Rue J, Vaysse F, Roméas A et al. (2007) Localization of STRO-1, BMP-2/-3/-7, BMP receptors and phosphorylated Smad-1 during the formation of mouse periodontium. Tissue Cell 39: 257-266. doi:10.1016/j.tice.2007.06.001. PubMed: 17662325. 17662325

[29] Fujita K, Iwasaki M, Ochi H, Fukuda T, Ma C et al. (2012) Vitamin E decreases bone mass by stimulating osteoclast fusion. Nat Med 18: 589-594. doi:10.1038/nm.2659. PubMed: 22388090. 22388090

