# Supplementary figures and images for: Comparative Gene-Expression Analysis of the Dental Follicle and Periodontal Ligament in Humans
Source: PLoS One. 2013 Dec 23;8(12):e84201. doi: 10.1371/journal.pone.0084201 (PMC3871683; doi:10.1371/journal.pone.0084201)

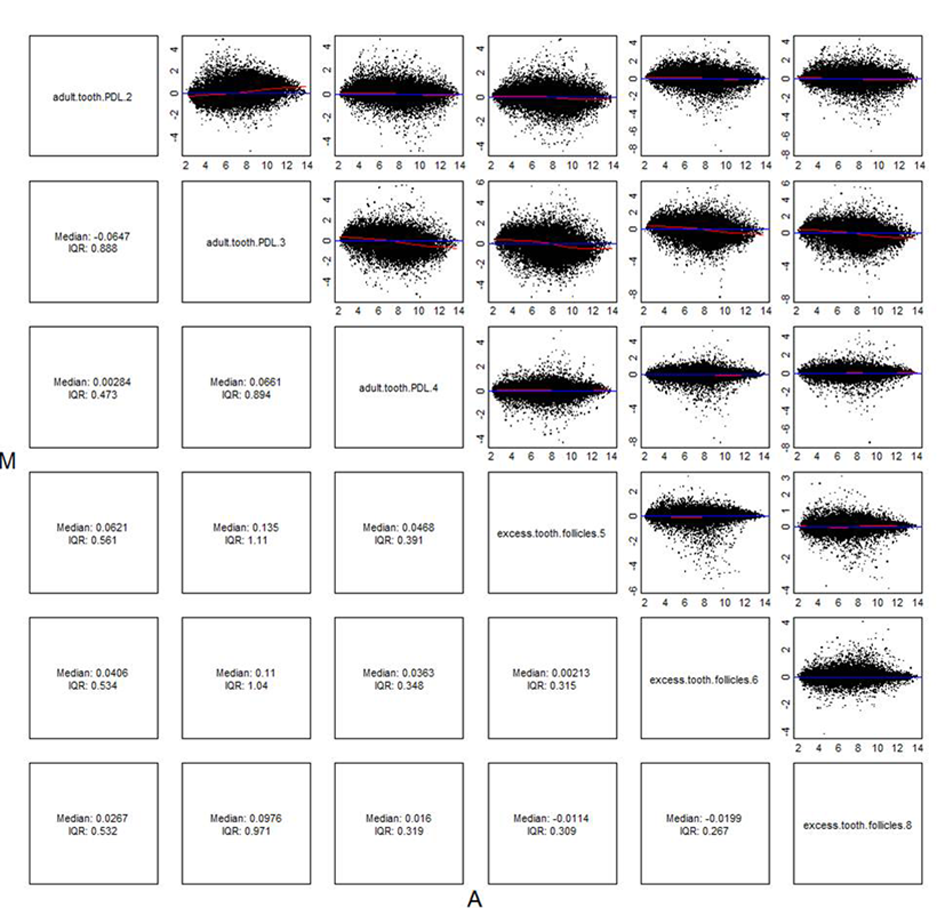

Supplement: Figure S1 — Scatter plots of the differences in the log intensities versus the average log intensity between three dental follicle tissue samples and three PDL tissue samples. In each plot the x-axis (A) is 0.5 × [log2(case)+log2 (control)] and the y-axis (M) is log2 (case/control). The data of all plots were normally distributed. (TIF) [file pone.0084201.s001.tif]
