# Supplementary material for: Comparative Gene-Expression Analysis of the Dental Follicle and Periodontal Ligament in Humans
Source: PLoS One. 2013 Dec 23;8(12):e84201. doi: 10.1371/journal.pone.0084201 (PMC3871683; doi:10.1371/journal.pone.0084201)
Supplement: Table S1 — Quantitative RT-PCR primers used in this study. (DOCX) [file pone.0084201.s002.docx]

Table S1. Quantitative RT-PCR primers used in this study

| Gene | Primer Assay ID | Product Size (bp) |
| --- | --- | --- |
| AMTN | Hs00418384_m1 | 62 |
| CD36 | Hs01567185_m1 | 116 |
| CXCL13 | Hs00757930_m1 | 70 |
| DMP1 | Hs01009391_g1 | 106 |
| EGFL6 | Hs00170955_m1 | 60 |
| MMP8 | Hs01029057_m1 | 67 |
| MMP9 | Hs00234579_m1 | 54 |
| WIF1 | Hs00183662_m1 | 72 |
| 18S rRNA | Hs03003631_g1 | 69 |
